# The Masquelet technique triggers the formation of a network involving LncRNA, circRNA, miRNA, and mRNA during bone repair

**DOI:** 10.1080/07853890.2024.2395591

**Published:** 2024-10-23

**Authors:** Muguo Song, Xiaoyong Yang, Xijiao Zhang, Junyi Li, Yongqing Xu, Jian Shi

**Affiliations:** Department of Orthopaedics, 920th Hospital of the Joint Logistics Support Force of the PLA, Kunming City, Yunnan Province, China

**Keywords:** Bone defects, Masquelet technique, lncRNA/circRNA-miRNA-mRNA ceRNA network

## Abstract

**Background:**

The ceRNA network, which is competitive endogenous RNA, uncovers a fresh mechanism of RNA interaction and holds significant importance in diverse biological processes. The aim of this study is to investigate the molecular process of induced membrane (IM) formation in bone defects using the Masquelet’s induced membrane technique (MIMT), in order to offer novel insights and a theoretical foundation for enhancing the treatment of bone defects with MIMT.

**Methods:**

In this work, we identified differentially expressed mRNAs (DEGs), lncRNAs (DELs), circRNAs (DECs), and miRNAs (DEMs). To explore the primary functions of the shared DEGs, we utilized Gene Ontology (GO) and Kyoto Encyclopedia of Genes and Genomes (KEGG) pathway enrichment analyses. Next, predictions were made for lncRNA-miRNA and miRNA-mRNA interactions, and the Cytoscape software was utilized to construct the regulatory network for ceRNA.

**Results:**

By integrating GO and KEGG enrichment analysis, a total of 385 differentially expressed genes (DEGs) were discovered in the samples from the MIMT-treated group. Additionally, after re-annotating the probes and intersecting two sets of differently expressed miRNAs, 1304 differentially expressed lncRNAs (DELs) and 23 differentially expressed circRNAs (DECs) were identified. Furthermore, 13 differentially expressed miRNAs (DEMs) were obtained. Moreover, utilizing the anticipated objectives of DEMs, we acquired 1203 pairs of lncRNA-miRNA-mRNA interactors (comprising 24 lncRNAs, 10 miRNAs, and 115 mRNAs) and 250 pairs of circRNA-miRNA-mRNA interactions (comprising 7 circRNAs, 9 miRNAs, and 115 mRNAs). CEBPA, DGAT2, CDKN1A, PLIN2, and CIDEC were identified as the five hub proteins in the PPI network. LncRNA/circRNA-hsa-miR-671-5p could potentially regulate the primary central protein, CEBPA.

**Conclusions:**

In this study, we described the potential regulatory mechanism of the MIMT in treating bone defects. We proposed a new lncRNA–miRNA–mRNA ceRNA network that could help further explore the molecular mechanisms of bone repair.

## 1. Introduction

Deficiency of long bones in limbs is one of the most common diseases in orthopedics [1,2]. The bone defect is a common and persistent disease in the orthopedic clinic, especially for those with skin and soft tissue defects at the same time, we should not only repair the defect of bone and skin and soft tissue but also solve the problem of bone infection [3]. There are various treatment methods, the clinical treatment of bone defects is mostly based on surgery, including Ilizarov technology, bone filling technology of autologous or allogeneic bone, filling technology of artificial bone replacement material, etc. Nevertheless, drawbacks such as donor-site morbidity and the restricted availability of grafts for harvesting render autografts a suboptimal choice for specific patient populations [4]. The utilization of Ilizarov technology typically impacts treatment efficacy, primarily attributed to nonunion at fracture ends and soft tissue entrapment resulting from infection. Prolonged treatment duration can also give rise to complications, including pressure sores and infections [5].

Masquelet’s induced membrane technique (MIMT) is a relatively new two-staged approach to reconstructing segmental bone defects caused by resection of infected, tumorous, or traumatically injured bone tissue [6]. MIMT has exhibited significant potential to transform the repair of critical-sized bone defects, boasting several advantages over alternative methods. This has resulted in a global surge in MIMT procedures over the past few decades [7–10]. Currently, the main disadvantage of MIMT is that it requires two phases of surgery and two interventions on the affected area, and extensive bone defects may even require two or more surgeries, which increases the difficulty of the surgery and the risk of infection [11]. During the process of repairing bone deficiency with the MIMT, the induced membrane (IM) plays the most important role [12].

The formation of IM involves multiple cellular processes, including a large number of mesenchymal stem cells (MSCs) [13,14]. Furthermore, in addition to creating an enclosed setting to impede the absorption of grafted bones and prevent the infiltration of fibroblasts and adipose tissue, IM also has the capability to release various growth factors. This, in turn, establishes a favorable environment for the development of blood vessels, stimulating the expansion and specialization of MSCs, consequently initiating the formation of new bone [15]. The formation of IM and the bone healing process not only influenced by osteoclasts, which participate in the regulation hematopoietic, bone formation regulation, regulation of blood vessel generation in bones, and hormone regulation of bone calcium [16]. But also macrophages participate in the bone resorption process in some localized inflammatory lesions [17]. During MIMT treat bone defects in IM formation, immune inflammation can affect MSCs proliferation and differentiation, and even directly affect the function of osteogenic cells and osteoclasts [18–21]. However, changes in gene expression in IM formation and the bone healing process are still unclear [22]. The absence of comprehensive knowledge poses a substantial challenge in refining the MIMT and enhancing success rates through technological advancements and precise patient selection [23]. Consequently, the limited understanding hampers broader adoption. Therefore, scrutinizing the alterations in various genes during IM formation in bone defects is imperative. Such analysis aids in elucidating the molecular mechanisms involved, thereby furnishing a theoretical foundation to enhance the prognostic outcomes of MIMT in bone defect treatment [24].

Transcripts known as competing endogenous RNAs (ceRNAs) have the ability to mutually regulate one another at the post-transcriptional level through competition for common miRNAs [25]. The function of protein-coding mRNAs is interconnected with non-coding RNAs such as microRNA, long non-coding RNA, pseudogenic RNA, and circular RNA through CeRNA networks [26–28]. Given that any transcripts containing miRNA response elements have the potential to act as ceRNAs, they might serve as a prevalent means of post-transcriptional control over gene expression in both normal biological processes and disease conditions.The activity of ceRNA is influenced by various elements, including the quantity and cellular distribution of ceRNA constituents, the strength of miRNA binding to their decoys, RNA modification, secondary structures of RNA, and RNA-binding proteins.

The objective of this study was to utilize bioinformatics techniques to examine the alterations in lncRNA, miRNA, and mRNA within the IM and adjacent bone tissue during the development of IM in individuals with bone defects. The aim was to construct a ceRNA regulatory network, identify significant ncRNA-miRNA-mRNA pathways, and investigate the mechanism behind IM formation in bone defects. These findings would provide a theoretical foundation for the application of MIMT technology in bone defect treatment.

## 2. Materials and methods

### 2.1. Data collection

For this research, we chose 3 individuals who had bone defects and were treated with MIMT in 920th Hospital of the Joint Logistics Support Force of the PLA during May 2022 to May 2023. We collected IM and bone tissue from the area of the bone defects 6 to 8 weeks after the surgery, which formed the study group (group T: T6: male, 52 years old; T12: female, 32 years old; T13: female, 35 years old). Additionally, we selected the excess periosteum and bone tissue that were removed during subtrochanteric osteotomy orthopedics of 3 children with congenital hip dislocation as the control group (group C: C1: female, 1 year and 6 months old; C9: female, 1 year and 6 months old; C10: female, 10 years old). Weigh the collected bone tissue, homogenize it in TRIzol, store it at − 80° C, extract RNA and DNase, and remove the residual TRIzol by precipitation with ammonium acetate. The NextGen sequencing core was utilized to analyze the processed samples for quality, and the Illumina HS 4000 platform was employed for performing the end reading of 50 base pairs. The UM Bioinformatics Core conducted the processing and analysis of data. The obtained sequences were compared with human reference sequences and analyzed for genomic transcript levels. 920th Hospital of the Joint Logistics Support Force of the PLA ethics committee granted approval for the study (approval no.:2020-064 (Science)-01). Written informed consent was obtained for all participants. In addition, this study obtained the written informed consent of the children’s parents, and we respected and protected the rights and interests of the child participants during the study.

### 2.2. Masquelet’s induced membrane technique (MIMT)

MIMT is a relatively new two-staged approach to reconstructing segmental bone defects caused by resection of infected, tumorous, or traumatically injured bone tissue.

Inclusion criteria:patients with tibial segmental bone defect length ≥4 cm and treated with induced membrane technique; patients with combined bone infections that have been effectively controlled, and those with exposed bone defects that have been repaired with flaps; patients with complete follow-up data. Exclusion criteria: serious peripheral nerve or vascular injury; lower extremity arterial and venous vascular thromboembolism or peripheral neuropathy; serious heart, lung, liver, kidney and other medical diseases or diabetes; a history of long-term hormone medication; psychiatric disease or do not cooperate with the treatment; follow up time <12 months.

Surgical methods and postoperative treatment: Bone defect end cleaning. Remove the free cortical bone block, bite off the sclerotic bone until the broken end oozes blood, open the medullary cavity. Bone cement filling, after infection control (6 weeks of postoperative antibiotic treatment, the symptoms of infection disappeared, and the blood test, erythrocyte sedimentation rate, C-reactive protein, etc. were normal for 3 times in the interval of 2 weeks), the second stage of the bone defect repair was carried out. The second stage of surgery (bone grafting) by the induced membrane technique was performed 7 to 9 weeks (average 8 weeks) after cement filling, and the amount of bone graft was 1.5 times the volume of the bone defect to be filled. The patient is instructed and supervised in active and passive flexion and extension of the knee, ankle, and interphalangeal joints from the second day after bone grafting. Two weeks after the operation, the patient is advised to start rehabilitation with crutches. Postoperative monthly review of the affected limb radiographs, to understand the healing of the end of the bone defect, according to the condition of the bone scab gradually weight-bearing, full weight-bearing after clinical healing; bone healing 2 ∼ 3 months after the review of the radiographs. Half a year after the bone healing, it is feasible to remove the fixation.

### 2.3. Detection of genes with altered expression: mRNAs, miRNAs, lncRNAs, and circRNAs

Using the limma package in the R software (version 3.3.2) [29,30], we identified lists of circRNAs (DECs), lncRNAs (DELs), miRNA (DEMs), and mRNAs (DEGs) that were differentially expressed between the Masquelet treatment groups and control groups samples. DEGs, DEMs, DECs, and DELs were screened using a significance level of *p* < 0.05, FDR < 0.05, and FC > 2.0. EPCLUST was employed for hierarchical clustering, representing the relationships among the samples based on the expression data matrix of differentially expressed genes (DEGs), differentially expressed transcripts (DECs), differentially expressed loci (DELs), and differentially expressed molecules (DEMs). Heat maps and volcano maps were generated using the ggplot and pheatmap packages in the R platform to display the DEGs, DECs, DELs, and DEMs.

### 2.4. Prediction of differentially expressed miRNAs includes identifying target genes, lncRNAs, and circRNAs

For the prediction of miRNA-mRNA target genes, three different algorithms were utilized: miRanda (http://www.microrna.org/), Targetscan (http://www.targetscan.org/), and miRWalk (http://129.206.7.150/). The predicted results consisted of the target genes that overlapped with genes identified through GO and KEGG pathway enrichment analyses. For the construction of the ceRNA network, the miRanda and PITA tools were utilized to forecast target lncRNAs, circRNAs, and those that intersected with DELs and DECs between Masqueret treatment groups and control groups. The prediction was based on the use of the miRanda and PITA tools, which can be found at https://genie.weizmann.ac.il/pubs/mir07/mir07_exe.html.

### 2.5. Construction of the ceRNA network

The ceRNA network, comprising lncRNA–miRNA–mRNA interactions, was assembled through the selection of miRNAs, lncRNAs, and mRNAs with altered expression levels. Specifically, we focused on those exhibiting inverse relationships with miRNAs in both the miRNA-mRNA and lncRNA-miRNA interaction pairs. This construction was grounded in the ceRNA theory, postulating that lncRNA functions as an endogenous ‘sponge’ to modulate mRNA expression by sequestering miRNA.

The presence of a miRNA-binding site in circRNA allows it to function as a miRNA-sponge, effectively competing with miRNA for binding and thereby preventing miRNA from regulating target genes, ultimately leading to indirect regulation of gene expression. By applying the ceRNA hypothesis, explore the circRNA-target gene pair that shares the identical miRNA binding site. Establish the circRNA-miRNA-mRNA regulatory connection, where circRNA acts as a decoy, miRNA as the central element, and mRNA as the target. This process leads to the formation of the ceRNA regulatory network. The ceRNA regulatory network unveiled the mechanism of ncRNA governing gene expression at the transcriptome-wide scale. Cytoscape software (version 2.8.2) was used to build and display the ceRNA network. Simultaneously, the calculation of node degrees for the lncRNA–miRNA–mRNA network was performed.

### 2.6. Performing enrichment analysis of differentially expressed genes (DEGs) using GO and KEGG pathways

The clusterProfiler R package was used to perform Gene Ontology (GO) enrichment analysis on differentially expressed genes, with correction for gene length bias.Differential expressed genes were considered significantly enriched by GO terms with P-values less than 0.05 that have been corrected. The KEGG database is a valuable resource for comprehending the overall functions and utilities of the biological system, including the cell, organism, and ecosystem. It provides insights into molecular-level information, particularly extensive molecular datasets produced by genome sequencing and other high-throughput experimental technologies. More information can be found at http://www.genome.jp/kegg/. To gain a deeper comprehension of the biological mechanisms of DEMs in the DELs-DEMs-DEGs network and DECs-DEMs-DEGs, we employed the clusterProfiler R package to e the statistical enrichment of differentially expressed genes in KEGG pathways.

### 2.7. PPI network analysis

The protein-protein interaction (PPI) network of the differentially expressed genes (DEGs) included in the competing endogenous RNA (ceRNA) network was constructed using the STRING database version 11.0. The PPI network was visualized using Cytoscape software (2.8.2), a cut-off value of > 0.4 for the combined score of protein pairs [31].

## 3. Results

### 3.1. Expression profiles of lncRNAs, circRNAs, miRNAs, and mRNAs

**Figure 1.**
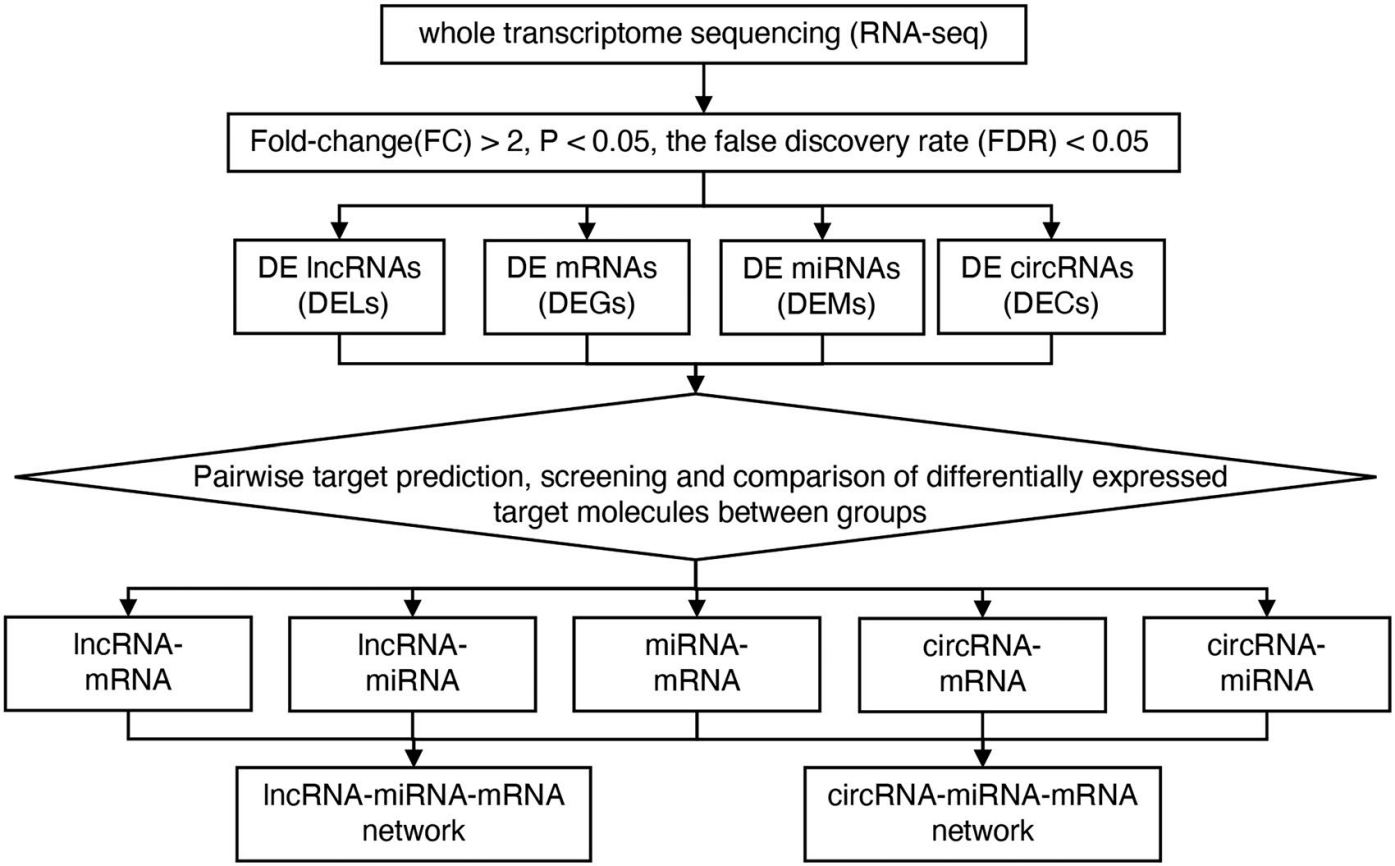
Flow chart of this study design.

The whole-transcriptome sequencing data (including lncRNA, circRNA, miRNA, and mRNA) were obtained from two distinct groups using the Illumina Hiseq 4000 platform. We conducted an analysis of differentially expressed (DE) non-coding RNAs (lncRNAs, circRNAs, miRNAs) and mRNAs with a cutoff for fold changes of ≥ 2 or ≤ 0.5, along with p-values < 0.05 and a false discovery rate (FDR) < 0.05 in both the study group (group T) and the control group (group C). The analytical strategy and procedures for the present study are illustrated in Figure 1.

### 3.2. Differential expression analysis

**Figure 2.**
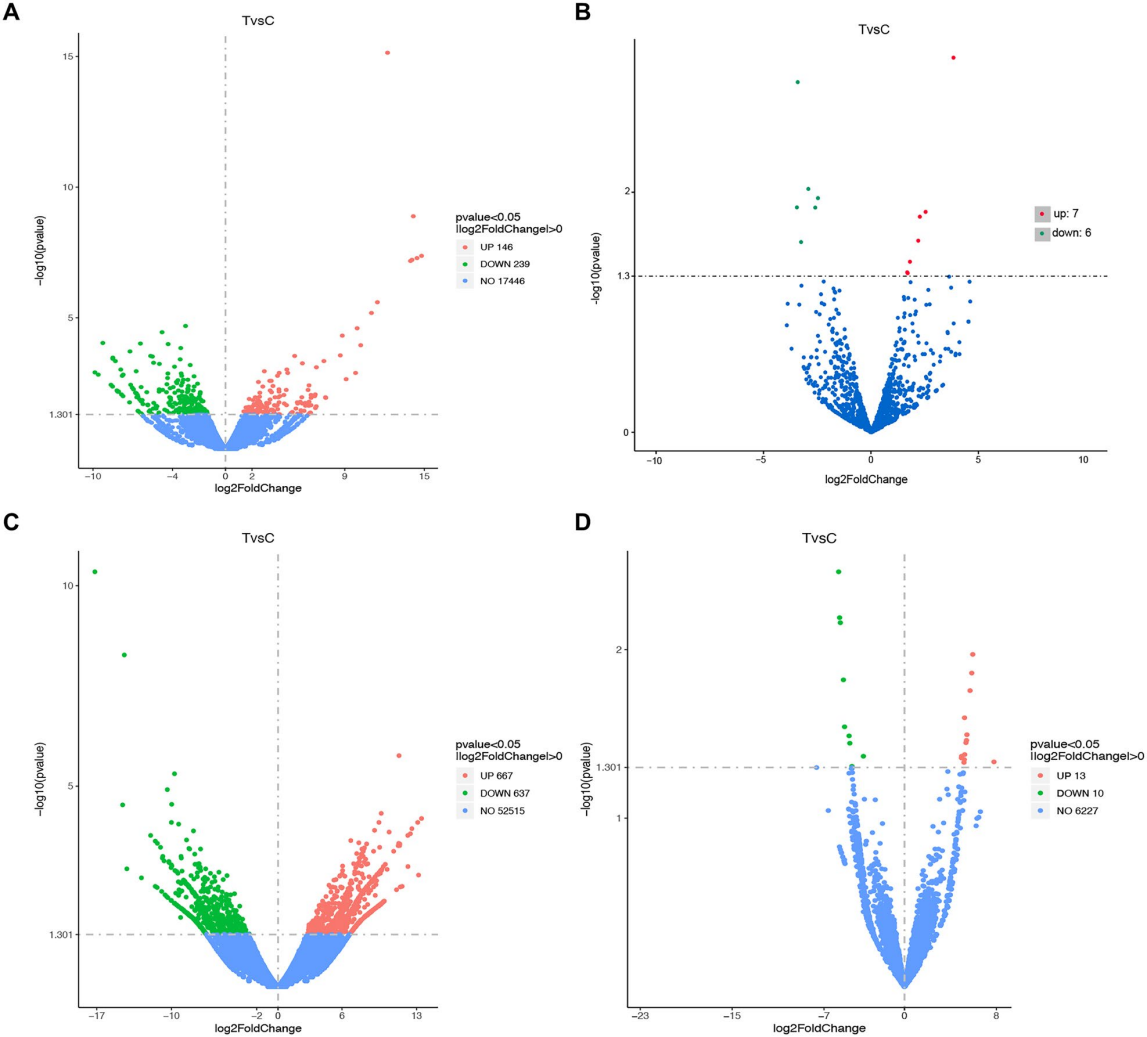
Volcano plot of DEGs, DEMs, DELs, and DECs. (A) Volcano Plot of differentially expressed mRNAs; (B) Volcano Plot of differentially expressed miRNAs; (C) Volcano Plot of differentially expressed lncRNA; (D) Volcano Plot of differentially expressed circRNAs. Upregulated genes are marked in light red; downregulated genes are marked in light green. (Differentially expressed with the cutoff fold changes ≥ 2 or ≤ 0.5 along with p < 0.05 and false discovery rate (FDR) < 0.05).

A total of 385 DEGs (146 upregulated and 239 downregulated), 13 DEMs (7 upregulated and 6 downregulated), 1304 DELs (667 upregulated and 637 downregulated), and 23 DECs (13 upregulated and 10 downregulated) were identified between the IM and bone tissue of bone defects treated with the MIMT at 6 to 8 weeks after surgery and control individuals (Figure 2A–D).

Table 1 illustrates that PARP1-213 exhibited the highest upregulation among the lncRNAs, showing a 13.50-fold increase, while RPL4-203 had the greatest downregulation with a 17.18-fold decrease. Additionally, the circRNA, miRNA, and mRNA that demonstrated the highest upregulation were hsa_circ_0001953 (with a 7.79-fold change), hsa-miR-671-5p (with a 3.82-fold change), and USP9Y (with a 14.80-fold change), respectively. The circRNA, miRNA, and mRNA that experienced the greatest decrease in expression were hsa_circ_0001971 (with a 5.73-fold change), hsa-miR-122b-5p (with a 3.47-fold change), and XLOC_058019 (with a 9.86-fold change), respectively.

**Table 1.** Statistical analysis of all of the differently expressed ncRNAs and mRNAs.

DE RNAs	Total No.	No. of Upregulated	No. of Downregulated	The Most Upregulated (Fold Change)	The Most Downregulated (Fold Change)
mRNA	385	146	239	USP9Y (14.80)	XLOC_058019 (9.86)
miRNA	13	7	6	hsa-miR-671-5p (3.82)	hsa-miR-122b-5p (3.47)
lncRNA	1304	667	637	PARP1-213 (13.50)	RPL4-203(17.18)
circRNA	23	13	10	hsa_circ_0001953 (7.79)	hsa_circ_0001971 (5.73)

**Figure 3.**
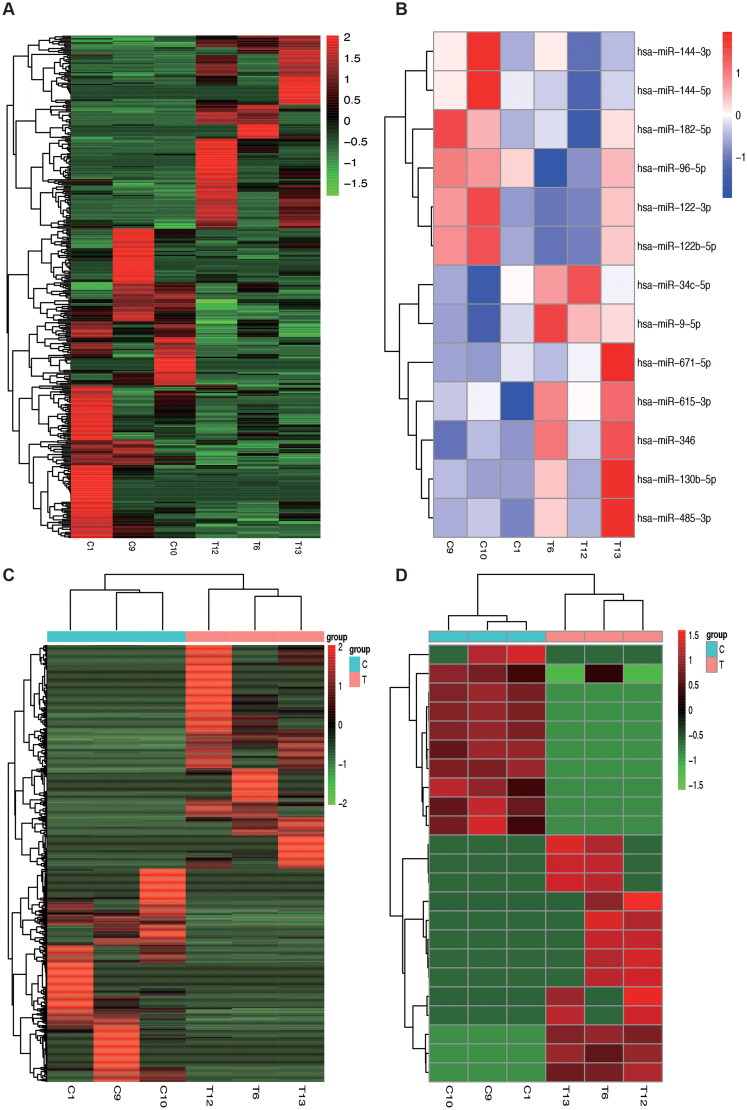
Heatmap analysis of DEGs, DEMs, DELs, and DECs. (A) Heatmap analysis of differentially expressed mRNAs; (B) heatmap analysis of differentially expressed miRNAs; (C) heatmap analysis of differentially expressed lncRNA; (D) heatmap analysis of differentially expressed circRNAs.

Supplementary materials, including Tables S1–S4, list the complete set of upregulated and downregulated DEGs, DEMs, DELs, and DECs. In conclusion, after applying the criteria of p-value < 0.05 and | log2(FC) | > 0.5, we generated heat maps for the DEGs, DEMs, DELs, and DECs, as displayed in Figure 3A–D.

### 3.3. GO and KEGG enrichment analysis of DEGs

**Figure 4.**
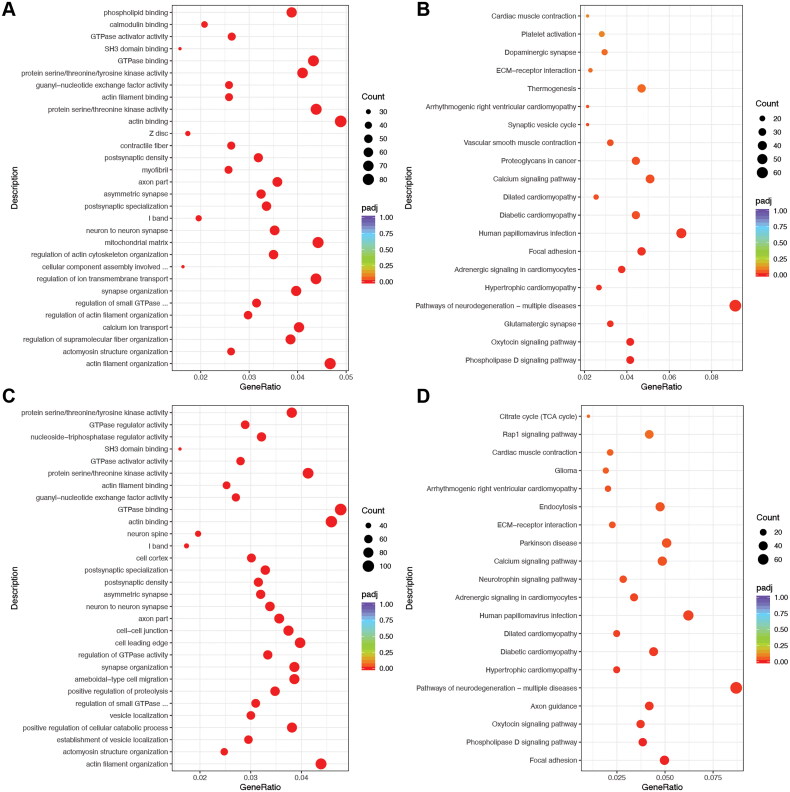
GO/KEGG enrichment analysis of DEGs. (A) The bubble plot of the top 30 enrichment pathways by DEGs in the lncRNA-miRNA-mRNA networks by GO analysis; (B) The bubble plot of the top 20 enrichment pathways by DEGs in the lncRNA-miRNA-mRNA networks by KEGG analysis; (C) The bubble plot of the top 30 enrichment pathways by DEGs in the circRNA-miRNA-mRNA networks by GO analysis; (D) The bubble plot of the top 20 enrichment pathways by DEGs in the circRNA-miRNA-mRNA networks by KEGG analysis.

Following the previously outlined analytical strategy, we constructed two intricate networks: the lncRNA-miRNA-mRNA network, consisting of lncRNA, miRNA, and mRNA, and the circRNA-miRNA-mRNA network, encompassing circRNA, miRNA, and mRNA. Given the abundance of relationship pairs, we conducted separate GO and KEGG pathway analyses for the mRNAs participating in the two intricate interaction networks. As depicted in Figure 4A, the GO analysis revealed that DEGs within the lncRNA-miRNA-mRNA networks were predominantly enriched in BPs such as ‘phospholipid binding’, ‘calmodulin binding’, and ‘GTPase activator activity’. The Cellular Component (CC) analysis highlighted significant enrichment in locations like the ‘Z disc’, ‘contractile fiber’, and ‘postsynaptic density’. Regarding Molecular Function (MF), the DEMs exhibited enrichment in processes like ‘regulation of actin cytoskeleton organization’, ‘cellular component assembly involved in morphogenesis’, and ‘regulation of ion transmembrane transport’. In parallel, the KEGG pathway analysis identified 20 pathways enriched in the DEGs of the lncRNA-miRNA-mRNA networks. Notably, signaling pathways such as ‘Cardiac muscle contraction’, ‘Platelet activation’, and ‘Dopaminergic synapse’ exhibited the highest enrichment (Figure 4B). Detailed information on the significant GO and KEGG pathways for the DEGs in the lncRNA-miRNA-mRNA networks can be found in the supplementary materials: Tables S5, S6.

As illustrated in Figure 4C, the GO analysis revealed that DEGs within the circRNA-miRNA-mRNA networks were predominantly enriched in BPs such as ‘protein serine/threonine/tyrosine kinase activity’, ‘GTPase regulator activity’, and ‘nucleoside − triphosphatase regulator activity’. The CC analysis highlighted significant enrichment in locations like the ‘neuron spine’, ‘I band’, and ‘cell cortex’. Regarding MF, the DEMs exhibited enrichment in processes like ‘regulation of GTPase activity’, ‘synapse organization’, and ‘ameboidal − type cell migration’. Concurrently, the KEGG pathway analysis identified 20 pathways enriched in the DEGs of the circRNA-miRNA-mRNA networks. Notably, the ‘Citrate cycle (TCA cycle)’, ‘Rap1 signaling pathway’, and ‘Cardiac muscle contraction’ signaling pathways exhibited the highest enrichment (Figure 4D). Detailed information on the significant GO and KEGG pathways for the DEGs in the circRNA-miRNA-mRNA networks is presented in the supplementary materials: Tables S7, S8.

### 3.4. Construction of bone defect repair related ceRNA network

It is well-known that the ceRNA regulatory mechanism plays a crucial role in the interaction between mRNA and ncRNAs, including miRNA, lncRNA, and circRNA. Applying the previously described analytical approach, we employed miRanda to predict the target mRNAs of these miRNAs. Subsequently, we conducted a comparative analysis between these target mRNAs and the differentially expressed mRNAs identified in our RNA-seq results. This study discovered 13 differentially expressed miRNAs (DEMs), with our primary focus on investigating whether these miRNAs could potentially target the 385 DEGs, 1304 DELs, and 23 DECs.

**Figure 5.**
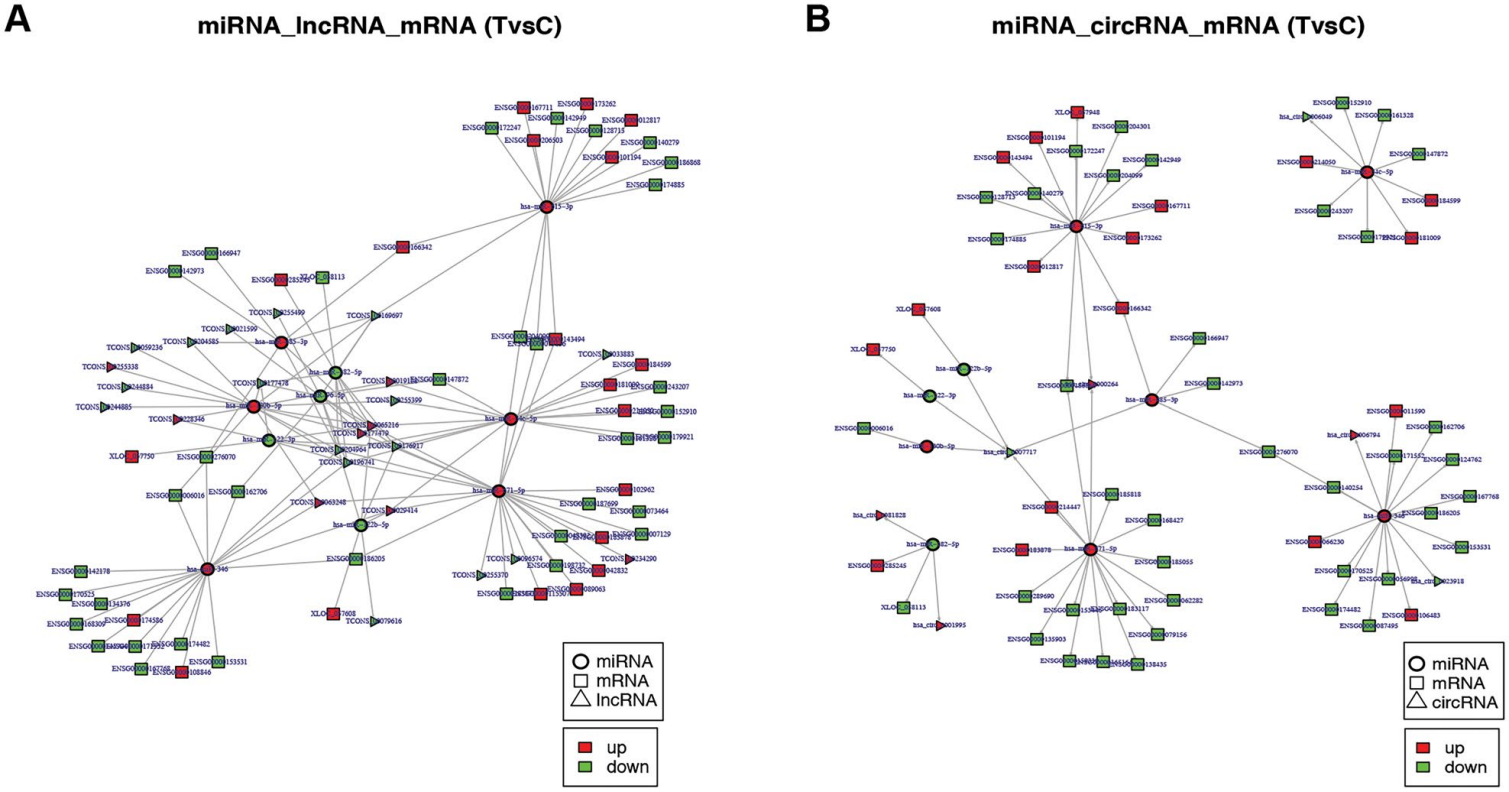
The analysis view of lncRNA, miRNA, mRNA target prediction, and ceRNA network. (A) lncRNA-miRNA-mRNA ceRNA network; (B) circRNA-miRNA-mRNA ceRNA network. The quadrilateral is miRNA, the triangle is lncRNA, and the circular is mRNA. Red: up-regulation, blue: down-regulation.

We obtained 1203 pairs of lncRNA-miRNA-mRNA interactors (comprising 24 lncRNAs, 10 miRNAs, and 115 mRNAs) and 250 pairs of circRNA-miRNA-mRNA interactions (comprising 7 circRNAs, 9 miRNAs, and 115 mRNAs) based on the predicted targets of DEMs. Following the ceRNA hypothesis, we utilized the common miRNA as a connecting point to identify mRNA, lncRNA, and circRNA that exhibit an inverse correlation with the expression of the targeted miRNA. This enabled us to establish a ceRNA network consisting of lncRNA-miRNA-mRNA and circRNA-miRNA-mRNA interactions. We evaluated the quantity of lncRNA-mRNA and circRNA-mRNA interactions by examining all potential lncRNA-mRNA and circRNA-mRNA interactions that shared miRNAs. The interaction pairs between lncRNA-miRNA and circRNA-miRNA were acquired using the hypergeometric test, with a P-value < 0.05 after adjustment. In conclusion, the aforementioned outcomes should be entered into Cytoscape to create and exhibit the ceRNA network diagram for lncRNA-miRNA-mRNA and circRNA-miRNA-mRNA (Figure 5A,B).

### 3.5. PPI network analysis

**Figure 6.**
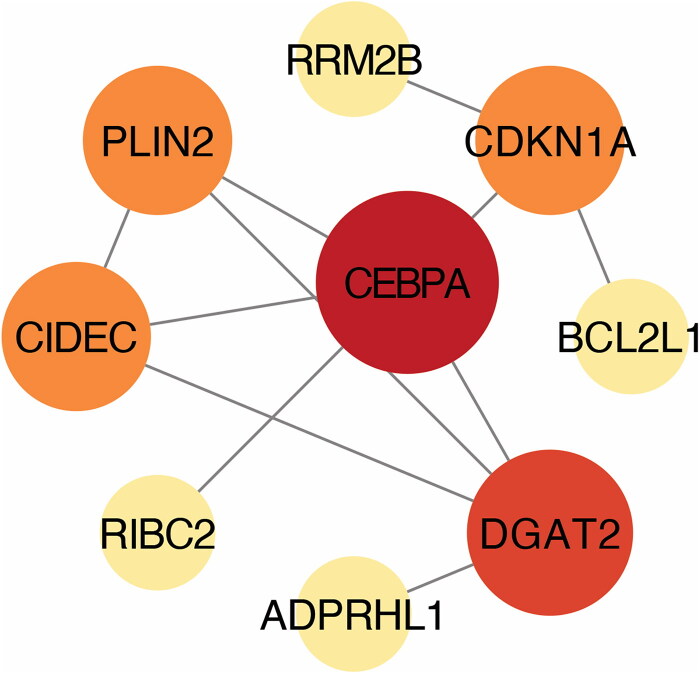
PPI Network and key genes analysis.

In order to further investigate the most important clusters of DEGs within the ceRNA network, we conducted a Protein-Protein Interaction (PPI) network analysis employing STRING database version 11.0 and visualized the outcomes through Cytoscape. Key hub proteins, namely CEBPA, DGAT2, CDKN1A, PLIN2, and CIDEC, emerged prominently (Figure 6, supplementary materials: Tables S9). Notably, the expression of CEBPA appears to be subject to regulation by multiple lncRNAs or circRNAs through hsa-miR-671-5p.

## 4. Discussion

The MIMT is a widely accepted and effective approach for the treatment of severe bone defects, particularly in the long bones of the extremities. The present study protocol aims to investigate the key factors involved in the process of IM formation after bone defects treated with MIMT, collect IM and bone tissue at the site of the bone defect were collected 6 to 8 weeks after surgery to comparing with excess periosteum and bone tissue cut during osteotomy and orthopedics in children. The protocol employs transcriptome sequencing to construct a ceRNA network and analyze the molecular mechanisms underlying IM formation. The study is expected to provide novel insights into the therapeutic potential of IM formation in bone defects. The time point for collect IM and bone tissue at the site of the bone defects was chosen to be within 6–8 weeks after treatment, as it has been established that the osteogenic and angiogenic activity of the induced membrane peaks during this period and gradually decreases thereafter [32]. The reason why excess periosteum and bone tissue removed during orthopaedic surgery in children was chosen as a control study in this study is that there is usually a lack of sufficient induced membrane in adults. The induced membrane is a membrane that plays a key role in fracture healing and bone regeneration by promoting the proliferation and differentiation of osteoblasts. The induced membrane elicits new bone vascularization and corticalization, and prevents graft resorption [33]. The excess periosteum and bone tissue cut during osteotomy and orthopedic surgery in children were selected for comparison in this study because is usually a lack of sufficient IM in adults, while Children have higher induced membrane activity than adults [34,35].

A total of 385 mRNAs that were differentially expressed were discovered through the implementation of a comparative analysis. Out of these, 146 mRNA molecules exhibited an upregulated expression profile, while 239 mRNA molecules demonstrated a downregulated expression pattern. USP9Y exhibited the highest upregulation with a 14.80-fold change, while XLOC_058019 showed the most significant downregulation with a 9.86-fold change. A total of 13 miRNAs showed differential expression, with 7 being upregulated and 6 being downregulated. The miRNA hsa-miR-671-5p exhibited the highest upregulation with a 3.82-fold change, while hsa-miR-122b-5p showed the greatest downregulation with a 3.47-fold change. The primary function of microRNA (miRNA) is to regulate gene expression at the post-transcriptional level by targeting the recognition of messenger RNA (mRNA) in the cell and inducing its degradation [36,37]. Research indicates a significant role for miRNA in the recuperation process of osteoporosis and bone injuries [38], contributing to diminished bone formation [39, 40]. These findings suggest the role of miRNA in bone healing, and exploring changes in miRNA during MIMT treatment and IM formation may provide effective targets for improving MIMT outcomes.

A grand total of 1304 LncRNAs exhibited differential expression, with 667 showing upregulation and 637 showing downregulation. Additionally, 23 circRNAs displayed differential expression, with 13 being upregulated and 10 being downregulated. The most upregulated lncRNA was PARP1-213, exhibiting a 13.50-fold change, while the most downregulated lncRNA was RPL4-203, showing a 17.18-fold change. Additionally, the most upregulated circRNA was hsa_circ_0001953 with a 7.79-fold change, and the most downregulated circRNA was hsa_circ_0001971 with a 5.73-fold change. LncRNA has a role in regulating epigenetics and can activate or interfere with transcription by binding with transcription factors, which is an important mechanism for regulating bone generation [41–43]. They can interact with transcription factors and bind to promoter regions, thereby regulating the expression of genes associated with bone healing [44–46]. Furthermore, lncRNAs can also exert post-transcriptional regulation [47,48]. Research indicates that the Wnt/β-catenin signaling pathway can be activated by LncRNA MEG3, leading to the enhancement of osteoblast cell proliferation and differentiation [49]. KCNQ1OT1 can also regulate FGFR3 expression by targeting miR-701-3p *via* ceRNA, thereby controlling osteoblast cell proliferation, migration, and apoptosis, providing a new therapeutic approach for fracture healing [50]. Furthermore, research has demonstrated a notable rise in the presence of lncRNA DANCR in the serum of individuals with fractures. Additionally, findings from cellular experiments conducted *in vitro* suggest that suppressing the DANCR level can considerably enhance the proliferation and differentiation of the MC3T3-E1 osteoblast cell line [51]. LncRNA HOTAIR can also regulate the fracture healing of osteoporotic rats by inhibiting miR-17-5p [52]. These studies all suggest the importance of lncRNA in the healing process of bones, but changes in lncRNA and miRNA during formation of IM during MIMT treatment have not yet been reported.

There is growing evidence that lncRNAs and miRNAs are differentially expressed and associated with a range of molecular processes involved in healing after bone injury, including regulation of osteogenesis, lipogenesis, and cell proliferation [38,48]. Despite this knowledge, the comprehensive regulatory network bridging the functionalities of coding and non-coding RNAs remains underexplored. This study employed transcriptome sequencing and bioinformatics analyses to scrutinize the GO and KEGG pathways enriched during the formation of the IM. Moreover, utilizing the anticipated objectives of DEMs, we acquired 1203 pairs of lncRNA-miRNA-mRNA interactors (comprising of 24 lncRNAs, 10 miRNAs, and 115 mRNAs) and 250 pairs of circRNA-miRNA-mRNA interactions (comprising of 7 circRNAs, 9 miRNAs, and 115 mRNAs). Subsequently, we constructed both lncRNA–miRNA–mRNA and circRNA–miRNA–mRNA ceRNA networks. The PPI network identified the five most significant hub proteins, with CCAAT enhancer binding protein alpha (CEBPA) being particularly noteworthy. CEBPA may be regulated by multiple lncRNAs or circRNAs through hsa-miR-671-5p. The CEBPA gene is responsible for regulating the growth inhibition and specialization of various cell types, including myeloid progenitors, adipocytes, hepatocytes, lung cells, and placental cells. Throughout the initial stages of embryogenesis, it performs crucial and overlapping roles alongside CEBPB—critical in the progression from CMP to GMP [53,54]. The excessive expression of miR-671-5p can impede the proliferation of osteosarcoma cells both *in vivo* and *in vitro* by obstructing cell cycle advancement [55].

It is important to acknowledge certain limitations in the present study. The RNA networks based on measurements predict a mechanism demonstrating the involvement of CEBPA, DGAT2, CDKN1A, PLIN2, and CIDEC in the formation of IM during the repair of bone defects through MIMT treatment. However, these predictions have not been validated through methods such as gene and protein expression analysis, and dual luciferase reporter gene analysis. While this research examines various interconnected genes for the initial time, additional clinical studies conducted *in vitro* and experiments conducted *in vivo* are necessary to validate their expression and functional mechanisms in the formation of IM.

There are certain strengths in this research work. Firstly, there is limited research on the role of cerna in IM development. This study is the initial investigation that builds ceRNA networks using IM and bone tissue for transcriptome sequencing, involving lncRNA-miRNA-mRNA and circRNA-miRNA-mRNA interactions. By analyzing the ceRNA networks, we can gain an in-depth understanding of the biological process of bone tissue repair, and provide a theoretical basis for the improvement of therapeutic methods. Secondly, based on the principle and clinical effect of MIMT, subsequent studies can be based on the ceRNA network of this study, combined with biomaterials, growth factors, or genetic engineering techniques, to improve the efficiency and quality of bone regeneration, thus promoting technological innovation and progress in the field of bone defect repair. Meanwhile, the present study identified a network of lncRNA/circRNA-hsa-miR-671-5p-CEBPA, which can be further validated to provide insights into the molecular mechanism and cellular signaling regulation of MIMT in the treatment of bone defects, and to provide theoretical support for the development of new therapeutic targets and individualized therapeutic strategies.

## 5. Conclusion

In this study, we described the potential regulatory mechanism of the MIMT in treating bone defects. The lncRNA/circRNA-hsa-miR-671-5p-CEBPA may play a key role in the IM formation during the MIMT treatment repair of bone defects. We proposed a new ceRNA network, this new lncRNA-miRNA-mRNA ceRNA network provides a novel perspective to understand the mechanism of MIMT in bone repair. Through the depth study of this ceRNA network, more molecular regulatory pathways and key genes can be revealed to help develop new therapeutic targets and individualized treatment strategies to improve the effect and success rate of MIMT in treating bone defects.

## Supplementary Material

Supplemental Material

## Data Availability

The datasets used or analyzed during the current study are available from the corresponding author on reasonable request.
